# Feeding Behavior, Water Intake, and Physiological Parameters of Feedlot Lambs Fed with Diets Containing Babassu Oil Associated with Sunflower Oil Blend

**DOI:** 10.1155/2024/8673922

**Published:** 2024-08-19

**Authors:** Laryssa V. da Silva, Gleice Kelle S. M. Vilela, Karlyene S. da Rocha, Hactus S. Cavalcanti, Glayciane C. Gois, Francisco Naysson de S. Santos, Fleming S. Campos, Michelle de O. M. Parente, Anderson de M. Zanine, Daniele de J. Ferreira, Tobyas M. de A. Mariz, Danielle de O. Maia, Henrique N. Parente

**Affiliations:** ^1^ Programa de Pós Graduação Em Ciência Animal Universidade Federal do Maranhão, Chapadinha 65500-000, MA, Brazil; ^2^ Programa de Pós-Graduação Em Zootecnia Tropical Universidade Federal do Piauí, Teresina 64049-550, PI, Brazil; ^3^ Departamento de Zootecnia Instituto Federal de Ciência e Tecnologia do Estado do Pará, Altamira 68377-630, PA, Brazil; ^4^ Departamento de Zootecnia Universidade Federal de Alagoas, Arapiraca 57309-005, AL, Brazil

## Abstract

This study aimed to investigate the impact of dietary inclusion of babassu oil (BO) associated with sunflower oil (SO) on feeding behavior, water intake, and physiological parameters of feedlot lambs. Thirty-five castrated male lambs (16.6 kg ± 3.9 kg) were distributed in a randomized block design with 5 treatments (diets) and 7 replications. The tested diets were oil-free diet (OF), 45 g/kg BO (BO), 30 g/kg BO with an additional 15 g/kg SO (1.5 SO), 22.5 g/kg BO with an additional 22.5 g/kg SO (2.25 SO), and 30 g/kg SO with an additional 15 g/kg BO (3.0 SO) on dry matter (DM) basis. The experimental period lasted 60 days. Animals that received BO diet and the combination of BO with SO had lower intakes of DM and neutral detergent fiber (NDF) compared to the control diet (*P* < 0.05). Differences on the respiratory rate (RR) was observed between animals in the control diet and those in the diets containing SO (*P*=0.001), with a linear increase in RR as the levels of SO in the diets increased (*P*=0.004). All physiological parameters showed a time effect (*P* < 0.05). Animals fed with the control diet had higher water intake via drinking fountain (*P*=0.030) and total water intake (*P*=0.029) compared to animals fed with diets containing SO. In relation to SO levels, water intake via drinking fountain (*P*=0.002), total water intake (*P*=0.002), and total water intake per kg of DM ingested (*P*=0.001) linearly increased with the levels increase in the composition of the diets. The tested diets did not alter the feeding behavior of the feedlot lambs. However, the combination of BO with different levels of SO reduced DM and water intake via drinking fountain and RR.

## 1. Introduction

Research using alternative sources of feed, such as byproducts derived from biodiesel, has been conducted to reduce production costs and meet the nutritional requirements of animals without harming their productive performance [1]. The use of vegetable oils in the diet of small ruminants can increase the energy density of the diet, reduce fermentation and calorific increment, and improve the productive efficiency of the animals [2]. This is especially significant in tropical regions where high environmental temperatures often negatively impact animal performance in confinement systems [3, 4], where animals often need to activate thermoregulatory mechanisms to mitigate stressors, directly affecting feed intake [5].

Vegetable oils are considered highly unsaturated sources and can, therefore, alter the metabolism of the microbial population in the rumen, consequently affecting thermoregulation and the feeding behavior of animals [1, 5]. Brazil has a great floristic diversity, including a large number of palm species with potential for vegetable oil production. The babassu palm (*Attalea speciosa* Mart. ex Spreng) is one of the main native palms in Brazil, primarily found in the states of the Northern and Northeastern regions, predominantly in the Amazon Rainforest region. Babassu is considered the largest global source of wild seed oil, accounting for approximately 72% of the almond weight [6].

Babassu oil (BO) mainly contains medium-chain fatty acids (MCFA), such as lauric acid (47.40%) and myristic acid (15.64%) [7, 8]. This composition of saturated fatty acids is highly relevant in tropical regions, as vegetable oils with a higher proportion of unsaturated fatty acids can be more vulnerable to oxidation [9]. The use of BO in the diet of small ruminants is primarily due to its physicochemical characteristics and its ability to serve as a source of readily available energy, mitigating thermal discomfort in regions with high temperatures [8]. This was observed by Machado et al. [5], who studied the physiological responses, feeding behavior, and water intake of lambs supplemented with BO or buriti oil in confinement. They found that using BO in diets adversely affects the animals' respiratory rate and water intake via drinking fountain, which are important physiological responses to thermal stress in lamb production in tropical regions.

Another vegetable oil widely used in animal feed is sunflower oil (SO). The sunflower (*Helianthus annuus* L.) is a plant native to North America and found in Brazil, especially in the south, southeast, midwest [10], and northeast [11] regions. Its seeds yield 52% oil [12], which has a low content of saturated fats (about 10%) [13] and is rich in linoleic acid (25.5–54.9% of total fatty acids). This acid can be converted into conjugated linoleic acid (CLA) by ruminants, explaining its high concentration in products derived from these animals [14].

In tropical conditions, the major challenge is to optimize animal performance by reducing the effects of heat stress in small ruminants [15]. Dietary effects can influence the ingestive behavior and physiological responses of animals as they adapt to nutritional changes [16]. Therefore, the balance of saturated and unsaturated fatty acids in diets requires comprehensive investigation, as it can mitigate the adverse impacts of vegetable oils when used alone in the offered feed composition [18, 19]. In this context, it is important to determine the ideal mixture of vegetable oils to improve animals' feeding behavior and thermoregulatory responses, especially in the face of climatic adversities.

Due to the great importance of nutrition in intensive production systems, research has shown the efficiency of babassu oil [7, 16, 17] and sunflower oil [20–22] in the diet of small ruminants, but separately. To the best of our knowledge, no studies have investigated the effect of combining BO with SO on feeding behavior, physiological parameters, and water intake of confined lamb.

Therefore, we hypothesize that combining BO with SO may contribute to the energy density of the diets, promoting a reduction in feeding frequency without affecting the lambs' physiological parameters and water intake. The aim of this study was to evaluate the dietary association of BO and SO in feedlot lambs, focusing on feeding behavior, water intake, and physiological parameters.

## 2. Materials and Methods

### 2.1. Location

Animal handling followed the guidelines recommended by the Animal Care and Use Committee of the same institution (process number 23115.009213/2019-23).

The experiment was conducted at the Small Ruminant Sector, Center of Chapadinha Science, Federal University of Maranhão, located in Chapadinha, MA, Brazil (3°44′26″ S, 43°21′33″ W, 104 m altitude). The tropical climate is classified as “Aw” according to Köppen [23], presenting a hot and rainy season from November to May, with an annual accumulated precipitation of 1670 mm and average annual temperature of 27°C [24]. During the experimental period (60 days), the minimum and maximum temperature and relative air humidity were recorded by a conventional station (Station Code: 82382) of the National Institute of Meteorology [25], obtaining average values of 20.8°C, 34.9°C, and 94%, respectively.

**Figure 1:**
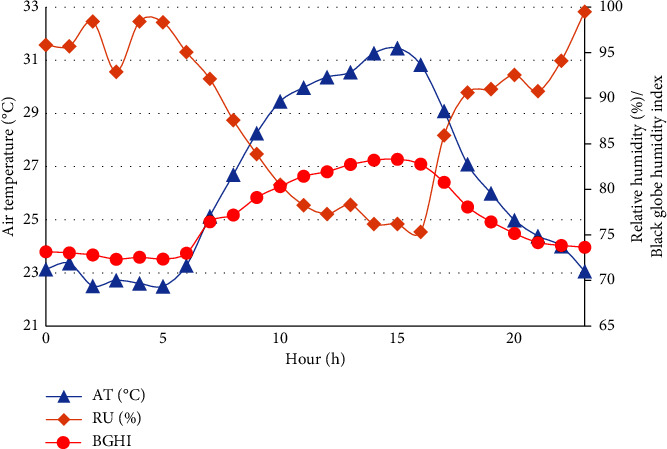
Average values of air temperature (AT), relative humidity (RH), and Black Globe Humidity Index (BGHI) obtained per hour during the experimental period (60 days).

The confinement was carried out in an open shed (without side walls), with a height of 3.5 meters, covered with metal roofing, and with a compacted dirt floor. During the experimental period, data of air temperature (AT) and relative humidity (RH) inside the confinement shed were collected using a digital thermohygrometer (Termômetro Higrometro, INCOTERM-7666.02.0.00, Franca, SP, Brazil). To determine the black globe humidity index (BGHI), the following equation was used [26] (Figure 1):

(1) $$\begin{array}{c} \text{BGHI}=\text{Tbg}+0.36\times \text{Tdp}+41.5, \end{array}$$

where Tbg = Black globe temperature and Tdp = dew point temperature.

**Figure 2:**
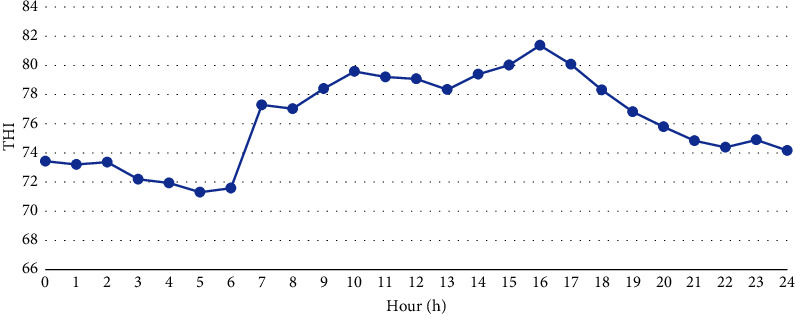
Average Temperature and Humidity Index (THI) values per hour during the experimental period (60 days).

The Temperature and Humidity Index (THI) (Figure 2) was determined according to Mader et al. [27], where

(2) $$\begin{array}{c} \text{THI}=0.8\times \text{AT}+\left(\left(\frac{\text{RH}}{100}\right)\times \left(\text{AT}-14.3\right)\right)+46.4. \end{array}$$

The thermal comfort/stress ranges observed were classified according to Silanikove and Koluman [28], who defined THI ranges as follows: <74 (comfortable), 75–79 (moderate stress), 80–85 (stressful), 86–88 (severe stress), and >88 (extreme stress). All animal handling was carried out by the same research team, remaining with the animals since the adaptation period. Therefore, during all analyzes the animals remained calm.

### 2.2. Animals and Experimental Facilities

Thirty-five noncastrated crossbred Dorper × Santa Ines lambs with an average initial body weight (IBW) of 16.6 ± 3.9 kg and an average age of 5 months were housed in individual pens (1.3 × 2.5 m) with a concrete floor and equipped with drinkers (10 L) and feeders (length: 30 cm, width: 26 cm, and height: 16 cm). The experiment lasted 60 days, with 10 days for the adaptation of the animals to the facilities and experimental diets. During the adaptation period, the animals were identified, weighed, and dewormed for endo and ectoparasites (Ivermectin® at a dose of 1 mL per 30 kg of BW).

### 2.3. Experimental Design, Treatments, and Dietary Management

A randomized block design was employed with five treatments and seven replications, resulting in 35 experimental units. Lambs were grouped into blocks based on their IBW at the beginning of the experiment. The treatments consisted of five diets as follows (dry matter basis): oil-free diet (OF, control), 45 g/kg BO (BO), 30 g/kg BO with an additional 15 g/kg SO (1.5 SO), 22.5 g/kg BO with an additional 22.5 g/kg SO (2.25 SO), and 30 g/kg SO with an additional 15 g/kg BO (3.0 SO).

The experimental diets (Table 1) were designed to be isonitrogenous and included oil blends with varying proportions of saturated FA (BO) and unsaturated FA (SO), aligned with the treatment specifications. The experimental diets were formulated in a roughage-to-concentrate ratio of 30 : 70 to obtain daily gains of 200 g, following the recommendations of the NRC [29].

In the diet formulation process, corn was coarsely ground using a grinder (Trapp, TRF 80, Jaragua do Sul, SC, Brazil) and combined with soybean meal, mineral premix, babassu oil, and/or sunflower oil following the treatments. The concentrate and Tifton-85 hay were separately weighed utilizing an electronic scale (Welmy, BCW 6/15/30, Santa Barbara d'Oeste, SP, Brazil) and provided as a total mixed ration once daily at 8 a.m.

### 2.4. Food Sampling

The amount of feed offered and refused was recorded daily to adjust the feed offered to ensure 10% of refusals. The animals had access to feed and water *ad libitum*. Dry matter intake (DMI) and neutral detergent fiber intake (NDFI) were obtained by the difference between the total dry matter (DM) of feed offered and the total DM and total NDF present in leftovers. Samples of the ingredients, diets, and refusals were collected daily and pooled by animal and then frozen at −20°C for further evaluations.

### 2.5. Feeding Behavior and Water Intake

Individual observations of the animals were carried out on the 31st and 52nd days of the experiment over 24 hours for better observation of the adaptation of animals to the diets offered. The animals' behaviors (feeding, ruminating, idling, and feeder visits; Table 2) were identified and recorded according to the methodology proposed by Martin and Bateson [30] using instantaneous and continuous sampling, using the focal sampling method and sampling intervals of 10 minute, with continuous periods of 24 h, starting at eight in the morning. The discretization of the time series was done by counting the discrete periods of feeding, ruminating, and idling. The average duration of each of the discrete periods was obtained by dividing the daily times of each activity by the number of discrete periods of the same activity [31].

Five trained observers recorded animal behavior data, minimizing interference whenever possible. Each observer was responsible for recording the activities of 7 animals (1 observer per treatment). The time spent on feeding, ruminating, and idling activities was obtained using digital timers. During the night-time observation data collection, the environment was kept under artificial lighting. For this, the animals underwent two days of adaptation to the artificial lighting.

Water was offered daily, at 7 : 30 am. Water was supplied in 10 L plastic buckets and weighed before delivery and weighed again after 24 h to determine water intake via drinking fountain (WID). Three buckets containing water were distributed in the shed, close to the stalls, to determine daily evaporation. Water samples were collected for laboratory analysis (Table 3).

For water intake via food (WIF) and total water intake (TWI), the values were calculated according to the following equations:

(3) $$\begin{array}{c} \text{WIF}=\text{intake of food in natural matter}-\text{dry matter intake}, \end{array}$$

(4) $$\begin{array}{c} \text{TWI}=\left(\text{water supplied}-\text{ water evaporated}\right)+\text{diet water}. \end{array}$$

Total water intake per kg of dry matter ingested (TWI.DMI) was also estimated.

Water intake was calculated as the difference between the daily supply and consumption, adjusted for average daily evaporation. Evaporation was determined by weighing containers filled with a known amount of water from one day to the next. These evaluations were conducted over a 5-day period and calculated as follows:

(5) $$\begin{array}{c} \text{WI}=\left(\text{QS}-\text{DO}\right)-\text{AE}, \end{array}$$

where WI = water intake; QS = quantity supplied; DO = daily ort; and AE = average daily evaporation.

### 2.6. Physiological Parameters

Physiological responses of the animals were assessed at 6:00 a.m., 10:00 a.m., 2:00 p.m., and 6:00 p.m. (one minute at each hour) over ten consecutive experimental days (from the 35th to the 44th day). Parameters including the respiratory rate (RR) and body and rectal temperatures (BT and RT, respectively) were measured. The RR (mov/minute) was determined through direct observation of left flank movements, according to Kawabata et al. [33], counting the number of movements during 15 seconds and the value obtained was multiplied by 4 to determine the RR in movements per minute. The BT (°C) was measured using a laser thermometer with an accuracy of ±0.8°C (Akrom model KR380, Porto Alegre, RS, Brazil), at a distance of 0.50 cm between the animal and observer. The average values were estimated as the average temperature of the snout, temple, back, and side of the lambs [5]. The RT (°C) was measured by inserting a veterinary clinical thermometer (Incoterm Termo Med 1.0, São Paulo, SP, Brazil) into the rectum for 2 minutes at a depth of 5 cm so that the bulb was in contact with the animal's mucosa.

### 2.7. Chemical Composition Analysis of Diets

The collected samples were ground using a 1 mm Wiley Mill screen (Marconi, Piracicaba, SP, Brazil) to determine the dry matter content (DM; Method 967.01), ash (Method 942.05), ether extract (EE; Method 954.05), and total nitrogen (N; Method 968.06) according to the AOAC [34]. Crude protein (CP) was calculated by multiplying the total nitrogen by 6.25. The neutral detergent fiber, assayed with a heat-stable amylase and expressed exclusively of residual ash (aNDFom), was determined following the Mertens [35] method. The total carbohydrates (TCs) were determined according to Sniffen et al. [36] and Mertens [35], respectively. The nonfiber carbohydrates (NFCs) were determined according to Hall [37].

### 2.8. Statistical Analysis

The data were analyzed using the MIXED model procedure in a regression analysis (SAS Institute Inc., Cary, NC), considering the diet as a fixed effect and the animal as a random effect. Bartlett's test was employed to assess the normality and homogeneity of variances for each variable [38]. Orthogonal contrasts were used to compare the control group with the diets containing BO or SO. The means were estimated using the LSMEANS statement, with a significance level (*α*) set at 0.05 for all analyses. The following mathematical model was used:

(6) $$\begin{array}{c} Yijk=\mu +Di+Ak+\varepsilon ijk, \end{array}$$

where *Yijk* is the response variable for the individual lamb, *µ* is the overall mean, *Di* represents the fixed effect of the diet, *Ak* represents the random effect of the animal, and *εijk* represents the random error.

Physiological parameters were analyzed using the MIXED model procedure (SAS Inst. Inc., Cary, NC) that took into account the same previous effects, with repeated measures over time, as described by the following mathematical model:

(7) $$\begin{array}{c} Yijk=\mu +Bi+Dj+Sij+Tk+\left(DT\right)jk+eijk, \end{array}$$

where *µ* = the overall mean; *Bi* = the random effect of block (*i* = 1–7); *Dj* = the fixed effect of diet (*j* = 1–5); *Sij* = the residual error associated with the animal effect (block × diet); Tk = the fixed effect of time (hours 6, 10, 14, and 18); (DT)*jk* = the interaction of the diet × time; and *Eijk* = the residual error. The most appropriate covariance structure selection was based on Akaike's Information Criteria Corrected (AICC) and Bayesian Information Criteria (BIC). The best model has the lowest AICC or BIC values. The covariance matrix that best fit the dataset was a first-order autoregressive-AR(1) for RR and BT and the first-order antedependence structure-ANTE(1) for RT.

For the feeding behavior, data were analyzed by PROC GLM procedure (SAS Inst. Inc., Cary, NC) and subjected to analysis of variance at a 5% probability level using the Tukey test. The following statistical model was used:

(8) $$\begin{array}{c} Yij=\mu +Bi+Dj+eij, \end{array}$$

where *µ* = the overall mean; *Bi* = the random effect of block (*i* = 1–7); *Dj* = the fixed effect of diet (*j* = 1–5); and *Eij* = the residual error.

## 3. Results

### 3.1. Environmental Variables

During the experimental period, the THI showed an increase starting at 7:00 a.m., remaining above 80 between 3:00 p.m. and 5:00 p.m., reaching 81.4 at 4:00 p.m., and subsequently declining (Figure 2).

### 3.2. Feeding Behavior and Water Intake

There was an effect of the diets on DM and NDF intakes (*P* < 0.05). Animals that received diets containing BO and the combination of BO with SO had lower intakes of DM and NDF, compared to the control diet. Feeding behavior did not show any treatment effects (*P* > 0.05) when comparing the control diet with the BO diet or the SO diets (Table 4).

Animals fed with the control diet had higher WID (*P*=0.030) and TWI (*P*=0.029) compared to those receiving the SO diets. About SO levels, it was observed that WID (*P*=0.002), TWI (*P*=0.002), and TWI.DMI (*P*=0.001) linearly increased with the levels increase in the composition of the diets. There was no effect of the tested diets on WIF (*P* > 0.05) (Table 4).

### 3.3. Physiological Parameters

There was a significant difference in RR between animals on the control diet and those on diets containing SO (*P*=0.001), with an increase in RR as the levels of SO in the diets increased (*P*=0.004) (Table 5). Lower RR was achieved with the inclusion of 15 g/kg of SO plus 30 g/kg of BO in the tested diets. For variables related to BT and RT, there was no effect of the diets (*P* > 0.05) (Table 5). All physiological parameters showed an effect of time (*P* < 0.05) relative to the treatments (Table 5).

The animals had lower RR during the morning, between 6:00 and 1000 a.m. (*P* < 0.001), and lower body BT (*P* < 0.001) and RT (*P* < 0.001) at 6:00 a.m. (Table 6).

## 4. Discussion

As expected due to the energy density, diets containing BO and BO and SO combination reduced the animals' DM and NDF intake. However, contrary to the hypothesis raised, diets containing BO and its combination with SO reduced RR, WID, TWI, and TWI.DMI, compared to the control diet. The composition of the diets may have influenced the responses obtained by reducing ruminal fermentation and calorific increment. Therefore, further research is necessary and relevant, involving the development of new diets with different levels of association between the tested oils or the use of oils in the composition.

### 4.1. Environmental Variables

The animals remained within the thermal comfort zone during the period from 00:00 to 06:00, with THI values below 74. According to Silanikove and Koluman [28], these data indicate thermal comfort. Between 07:00 a.m. and 02:00 p.m. and from 06:00 p.m. onwards, the animals experienced moderate stress [28], with THI values between >74 and 79, and severe stress during the period from 03:00 to 05:00 p.m. When observing BGHI (Figure 2), we can corroborate the findings for THI, as BGHI values were above 80 between 10:00 a.m. and 05:00 p.m., which is considered above the thermally neutral zone for adult sheep in dryland regions, according to Oliveira et al. [39]. These factors may have influenced the animals' physiological responses to environmental variables, indicating a time effect (Tables 5 and 6). This underscores the importance of establishing indicators of thermal discomfort to implement measures that mitigate animal stress without compromising productive variables.

### 4.2. Feeding Behavior and Water Intake

The lower intake of DMI and NDF by animals fed the BO diet and diets containing SO, compared to the diet without oil, may be related to the fact that these diets were more energy dense. The control diet contained 2.7% EE, while the other diets contained, on average, 6.9% (Table 1), due to the presence of oils, which may have limited DMI by the animals to meet their nutritional energy needs, generating a satiety effect earlier than the control treatment. Yamamoto et al. [40], studying sources of vegetable oils in the diet of confined lambs, reported that diets with higher energy content limit intake, corroborating with our findings.

In addition, it should be noted that among the tested diets, the BO diet resulted in the lowest intake of DM and NDF. This can be attributed to the nutritional properties of BO, which is rich in medium-chain fatty acids [17, 41]. Since these fatty acids are saturated and have a lower molecular weight [42], they are absorbed more quickly, increasing the animal's metabolism and leading to lower consumption [43]. It is also worth mentioning that lauric acid (C12 : 0) is the predominant saturated fatty acid in babassu oil (18.29%; Table 1), which may have increased the production of cholecystokinin, reducing feed intake by inhibiting gastric emptying, consequently decreasing motility and the rate of digestion through the gastrointestinal compartments [44]. On the other hand, the higher acceptability of diets containing SO may be related to its organoleptic characteristics and composition, being rich in polyunsaturated fatty acids. These fatty acids undergo the process of ruminal biohydrogenation to become saturated for absorption [45], making them more acceptable to the animals.

The lack of difference in feeding behavior can likely be attributed to the diets' similarity in terms of protein and NDF content and their nutritional adequacy for supporting productive performance. These mechanisms can influence food digestion and its passage rate through the gastrointestinal tract of ruminants. However, animals can adjust their ingestive behavior by modifying one or more components to overcome intake limitations and obtain the required nutrient quantity [46].

The greater water intake via drinking fountain and the total water intake by animals that received the control diet, compared to animals that received diets with SO, can be attributed to the higher energy concentration in the last ones, combined with the greater intake of dry matter that the animals subjected to this treatment presented. The lipids allow animals to quickly meet their energy needs due to the high caloric density of the diets [47], which leads to a lower DM intake and, consequently, a lower water intake. Thus, diets containing SO provided a limiting effect on animals, reducing dry matter intake, resulting in lower water intake.

### 4.3. Physiological Parameters

The higher RR was observed in animals receiving the control diet, likely due to their higher dry matter intake. Diets with high energy content limit dry matter intake, possibly reducing metabolism and lowering respiratory rates [4, 48]. According to Neiva et al. [49], the diet type significantly influences the susceptibility of the animals to environmental effects, even for hairless breeds originating from tropical regions. Therefore, interactions among the feed type, intake, environment, and physiological parameters should be considered to improve animal performance [48, 50]. The animals RR ranged from 34.0 to 44.0 mov/minute, which is above to normal values (20–34 mov/minute) of those established by Reece [51]. This increase in RR can be justified to the calorific increment from fermentation, digestion, absorption, and metabolism, stimulating elevations in this physiological response to maintain homeothermy [52].

The increase in RR is highly effective for heat dissipation, as the greater volume of air inspired/exhaled by the animal cools and moistens it, leading to increased heat loss through evaporation [53]. However, rapid and continuous breathing can add endogenous heat and divert energy that could be used in other metabolic and productive processes [4]. Therefore, in environments with high temperatures, the inclusion of 15 g/kg of SO plus 30 g/kg of BO may contribute to animal thermoregulation, as lower RR was observed in animals consuming this diet, indicating reduced endogenous heat production. In addition, this diet resulted in lower water intake for the animals, possibly due to increased metabolic water formation from nutrient oxidation. This diet can be recommended for regions with high temperatures and water scarcity. However, the energy expenditure required for the animal to maintain homeothermy may lead to a reduction in daily weight gain [54], requiring additional studies to evaluate the effect of the tested diets on the productive performance of confined lambs, in addition to a greater number of animals so that feeding behavior can be investigated with greater accuracy.

The minimal increase observed in BT (35.02–35.65°C) and RT (39.15–39.45°C) indicates that excess heat was efficiently dissipated through the animals' inherent thermoregulatory mechanisms [55]. Oliveira et al. [39] studied the effect of diets with high and low energy density on the physiological parameters of lambs and found similar values to this study for rectal temperature (39.16–39.25°C). However, the authors observed that the lambs had lower body temperatures (31.47–31.98°C) than we observed, which may be related to the location where the experiment was conducted and the environmental conditions (Brazilian Cerrado, which has an Aw type climate-seasonal tropical, characterized by dry winters and rainy summers, with temperatures reaching up to 39°, and with a thermal sensation of 42°) [23].

## 5. Conclusions

The used of BO and SO in lamb's diets reduced DMI and water intake via drinking fountain but did not alter the feeding behavior of the confined animals. The combination of 30 g/kg BO with 15 g/kg SO was the diet that caused a greater decreased respiratory rate

## Figures and Tables

**Table 1:** Proportion of the ingredients and chemical composition of the experimental diets.

Item	Diets				
	OF	BO	1.5 SO	2.25 SO	3.0 SO
Ground corn (%)	45.0	40.5	40.5	40.5	40.5
Soybean meal (%)	23.0	23.0	23.0	23.0	23.0
Tifton-85 hay (%)	30.0	30.0	30.0	30.0	30.0
Babassu oil (%)	0.0	4.5	3.0	2.25	1.5
Sunflower oil (%)	0.0	0.0	1.5	2.25	3.0
Mineral premix (%)	2.0	2.0	2.0	2.0	2.0
Chemical composition					
Dry matter (g/kg FM)	893	898	898	898	898
Crude protein (g/kg DM)	178	174	174	174	174
Neutral detergent fiber (g/kg DM)	407	398	398	398	398
Nonfibrous carbohydrates (g/kg DM)	333	308	307	309	302
Total carbohydrates (g/kg DM)	734	705	704	705	702
Ether extract (g/kg DM)	27	69	69	69	69
Metabolizable energy (Mcal/kg)	3.07	3.16	3.29	3.27	3.25
Fatty acid profile (g/100 g fatty acids)					
Caprylic acid (C8 : 0)	0.11	1.48	1.07	0.93	0.65
Capric acid (C10 : 0)	0.00	1.36	0.98	0.84	0.58
Lauric acid (C12 : 0)	0.84	18.29	12.74	10.92	7.36
Myristic acid (C14 : 0)	0.27	5.64	4.01	3.43	2.34
Palmitic acid (C16 : 0)	17.80	15.13	15.33	15.09	14.79
Stearic acid (C18 : 0)	10.90	9.96	10.59	10.56	10.59
Oleic acid (C18 : 1 *cis* 9)	24.02	19.36	20.49	20.60	21.34
Vaccenic acid (C18 : 1 *trans* 11)	1.02	0.72	0.78	0.76	0.77
Linoleic acid (C18 : 2 *n*6)	37.15	21.44	27.36	30.39	34.95
Linolenic acid (C18 : 3 *n*3)	5.61	4.59	4.54	4.34	4.36
Arachidic acid (C20 : 0)	0.86	0.70	0.74	0.75	0.76
Behenic acid (C22 : 0)	0.65	0.55	0.64	0.66	0.67
Lignoceric acid (C24 : 0)	0.76	0.67	0.72	0.72	0.73

OF: oil-free diet (control); BO: 45 g/kg BO; 1.5 SO: 30 g/kg BO with an additional 15 g/kg SO; 2.25 SO: 22.5 g/kg BO with an additional 22.5 g/kg SO; 3.0 SO: 30 g/kg SO with an additional 15 g/kg BO; FM: fresh matter; DM: dry matter.

**Table 2:** Description of the variables used to evaluate the feeding behavior of lambs fed with diets containing vegetal oils with different fatty acid profile.

Feeding behavior	Description
Feeding (min/day)	Eating; while the feed is still in the mouth
Ruminating (min/day)	Chewing the regurgitated feed, standing or lying down
Idling (min/day)	Standing or lying down without any movement or behavior
Feeder visits (*n*°)	Number of times the animal goes to the trough to feed

Adapted from Costa et al. [32].

**Table 3:** Analysis of the water offered to the animals during the experimental period.

Items	Results	Standards
Odor	Not objectionable	Not objectionable
Flavor	Not objectionable	Not objectionable
pH	7.8	6.0–9.0
Temperature	18.5	—
Turbidity	0.18	5 NTU
Chlorides	43.0	250 mg/L
Iron	0.05	0.3 mg/L
Total hardness	54.0	300 mg/L
Sulfate	71.0	250 mg/L
Nitrate	1.1	1.0 mg/L
Nitrite	0.010	1.0 mg/L
Free residual chlorine	1.25	0.2–5.0 mg/L
Apparent color	5.0	15 Hu
Electrical conductivity	785.0	*μ*s/cm

pH: hydrogenionic potential; NTU: nephelometric turbidity units; Hu: Hazen unit.

**Table 4:** Feeding behavior and water intake of lambs fed with diets containing vegetal oils with different fatty acid profile.

Items	Diets					SEM
	OF	BO	1.5 SO	2.25 SO	3.0 SO	
Dry matter intake	845.09	531.37	675.94	734.66	713.72	28.12
Neutral detergent fiber intake	355.05	229.15	307.71	322.06	313.28	11.97
Feeding (min)	161.41	188.91	178.69	203.58	214.61	9.95
Ruminating (min)	361.45	376.21	383.06	448.44	385.09	13.95
Idling (min)	918.96	875.06	878.42	787.98	840.30	20.33
Feeder visits (n°)	12.58	15.67	13.00	18.50	15.21	0.67
WID (kg/d)	2.72	1.71	1.54	1.85	2.01	0.13
WIF (kg/d)	0.11	0.08	0.09	0.12	0.09	0.005
TWI (kg/d)	2.83	1.79	1.63	1.97	2.10	0.13
TWI.DMI (kg/d)	3.05	2.72	2.18	1.91	2.73	0.11

	*P*-value					
	OF × BO	OF × SO	*L*	*Q*		

Dry matter intake	<0.001	<0.001	<0.001	0.011		
Neutral detergent fiber intake	0.152	<0.001	<0.001	0.041		
Feeding (min/day)	0.683	0.473	0.184	0.530		
Ruminating (min/day)	0.868	0.644	0.460	0.214		
Idling (min/day)	0.952	0.517	0.257	0.521		
Feeder visits (*n*°)	0.173	0.125	0.482	0.814		
WID	0.675	0.030	0.002	0.409		
WIF	0.567	0.085	0.208	0.802		
TWI	0.694	0.029	0.002	0.436		
TWI.DMI	0.089	0.113	0.001	0.138		

OF: oil-free diet (control); BO: 45 g/kg BO; 1.5 SO: 30 g/kg BO with an additional 15 g/kg SO; 2.25 SO: 22.5 g/kg BO with an additional 22.5 g/kg SO; 3.0 SO: 30 g/kg SO with an additional 15 g/kg BO; WID: water intake via drinking fountain; WIF: water intake via food; TWI: total water intake; TWI.DMI: Total water intake per kg of dry matter ingested; OF × BO: orthogonal contrast between the oil-free and babassu oil diets; OF × SO: orthogonal contrast between the oil-free and sunflower oil diets; SEM: standard error of mean; *L*: linear effect; *Q*: quadratic effect. Significant at the 5% probability level.

**Table 5:** Respiratory rate (RR, mov/min), body temperature (BT, °C), and rectal temperature (RT, °C) of lambs fed with diets containing vegetal oils with different fatty acid profile.

Items	Diets					SEM
	OF	BO	1.5 SO	2.25 SO	3.0 SO	
RR	43.99	35.67	33.96	41.86	40.28	0.96
BT	35.18	35.20	35.46	35.65	35.02	0.16
RT	39.45	39.25	39.15	39.27	39.31	0.04

	*P*-value					
	OF × BO	OF × SO	*L*	*Q*	*H*	

RR	0.560	0.001	0.004	0.306	<0.001	
BT	0.183	0.256	0.198	0.875	<0.001	
RT	0.517	0.126	0.064	0.894	<0.001	

OF: oil-free diet (control); BO: 45 g/kg BO; 1.5 SO: 30 g/kg BO with an additional 15 g/kg SO; 2.25 SO: 22.5 g/kg BO with an additional 22.5 g/kg SO; 3.0 SO: 30 g/kg SO with an additional 15 g/kg BO; OF × BO: orthogonal contrast between the oil-free and babassu oil diets; OF × SO: orthogonal contrast between the oil-free and sunflower oil diets; SEM: standard error of mean; *L*: linear effect; *Q*: quadratic effect; *H*: time effect. Significant at the 5% probability level.

**Table 6:** Effect of different observation times on respiratory rate (RR, mov/min), body temperature (BT, °C), and rectal temperature (RT, °C) of lambs fed with diets containing vegetable oils with different fatty acid profiles.

Items	Times				SEM	*P* value
	6:0 a.m.	10:00 a.m.	2:00 p.m.	6:00 p.m.		
RR	27.60b	44.16a	45.40a	46.14a	3.00	<0.001
BT	32.67b	35.74a	37.25a	35.56a	1.78	<0.001
RT	38.82b	39.22a	39.45a	39.49a	1.40	<0.001

SEM: standard error of mean. Means followed by different letters differ from each other using Tukey's test at a 5% probability level.

## Data Availability

The data used to support the findings of this study are available on request from the corresponding author.
